# A capless hairpin-protected mRNA vaccine encoding the full-length Influenza A hemagglutinin protects mice against a lethal Influenza A infection

**DOI:** 10.1038/s41434-025-00521-0

**Published:** 2025-02-23

**Authors:** Victor Solodushko, Jin H. Kim, Brian Fouty

**Affiliations:** 1 Department of Pharmacology, University of South Alabama School of Medicine, Mobile, AL USA; 2 The Center for Lung Biology, University of South Alabama School of Medicine, Mobile, AL USA; 3 Department of Microbiology & Immunology, University of South Alabama School of Medicine, Mobile, AL USA; 4 Department of Internal Medicine, University of South Alabama School of Medicine, Mobile, AL USA; 5 The Division of Pulmonary and Critical Care Medicine, University of South Alabama School of Medicine, Mobile, AL USA

**Keywords:** Nucleic-acid therapeutics, Drug delivery

## Abstract

The success of mRNA vaccines in controlling the COVID 19 pandemic has confirmed the efficacy of synthetically synthesized mRNA in humans and has also provided a blueprint on how to design them in terms of molecular structure and cost. We describe a mRNA vector that, unlike linear mRNAs used in current vaccines/therapeutics, does not require a 5′ cap to function. The described mRNA vector initiates translation from an internal ribosomal entry site (IRES) and contains specially designed self-folding secondary structures (hairpins) to protect the 5′ end against degradation, dramatically improving its stability. The produced mRNA did not require any additional modifications for functionality. The 5′ hairpins completely inhibited cap-dependent translation, and all vectors containing them required an IRES to express protein. When this capless mRNA vector was constructed to express the full-length Influenza A membrane protein hemagglutinin (HA), complexed with pre-formed lipid-based nanoparticles, and then injected into mice as a vaccine, it generated high titers of anti-HA antibodies and protected mice against a lethal dose of Influenza A.

## Introduction

The non-integrative nature and transient expression of mRNA makes it ideal for use as a vaccine. mRNA-based vaccines can induce both humoral and cell-mediated immunity and were the first vaccines against SARS-CoV-2 approved in the United States [1]. They have also shown promise in protecting against Influenza A in both a mouse model and a human clinical trial [2–5]. Because mRNA vaccines can be synthesized in cell-free conditions using a DNA template, they are less expensive and faster to produce than subunit, live-attenuated, and inactivated virus vaccines while avoiding the safety issues intrinsic to working with live viruses. This permits simpler downstream purification and rapid manufacturing [6]. mRNA vaccines have the potential to decentralize vaccine production and be a rapid effective way to control emerging infectious threats throughout the world [7, 8].

Almost all endogenous eukaryotic mRNA contains a 5′ cap which is added during RNA processing [9, 10]. The 5′ cap, an N7-methylated guanosine linked to the first nucleotide of the RNA via a reverse 5′ to 5′ triphosphate linkage (the 5′ m^7^G cap), is important in transcript splicing, nucleocytoplasmic transport, protein translation, and protection against 5’ exonucleases [11]. Most mRNA synthesized in vitro also contains a 5′ cap, placed there either during transcription using a variety of anti-reverse cap analogs or CleanCap® capping technology or post-transcriptionally using capping enzymes—e.g., from vaccinia virus [12]. When mRNA is produced exogenously and then delivered to cells as a vaccine or therapeutic, however, there is no need for a 5’ cap to splice the transcript or help transport it out of the nucleus; only initiation of translation and protection against exonucleases is required. Redesigning mRNA so they retain their function and stability without the requirement for a cap would reduce the cost, increase the speed, and improve the yield, of in vitro mRNA production.

Internal Ribosomal Entry Sites (IRES) are highly structured regions found in some mRNAs in which ribosomes can initiate translation [13]. IRES were originally identified in viruses, but have also been identified in eukaryotic mRNA [14–16], and can initiate translation when cap-independent translation is preferred or cap-dependent synthesis is shut down in response to infection or inflammation [17, 18]. IRES sequences are widely used in molecular biology to co-express additional genes from the same capped mRNA [19], but are not commonly used to initiate translation of the first gene or a single gene. The idea of synthesizing mRNA that harbors an IRES on the 5′ end without the need to add a cap is attractive due to its simplicity and cost efficiency. The main drawback of such an approach, however, is the extreme sensitivity of the 5′ end of unprotected mRNA to exonucleases making it unstable and highly degradable.

Previously, we addressed the problem of decreased transcript expression of capless and tailless mRNA by redesigning its ends to self-fold into triple hairpin structures, thus burying the 5′ and 3′ ends deep into heavily structured terminuses. This capless/tailless mRNA vector maintained a high level of reporter expression in eukaryotic cells if an encephalomyocarditis virus (EMCV) IRES was included in its 5′ untranslated region (UTR) [20]. These (hairpin and IRES) sequences can be included in the DNA template, meaning that the transcribed mRNA does not require any additional modifications, dramatically simplifying its production. We have found that the effect of these terminal structures on mRNA functionality is not reliant on a particular sequence and depends primarily on the stability of the hairpins formed.

Here, we describe a capless mRNA vector that includes a double (instead of a triple) hairpin at the 5’ end and encodes the full-length hemagglutinin (HA) of the Influenza A virus. When complexed with pre-formed lipid-based nanoparticles and used as a vaccine, it elicited a strong anti-HA antibody response and protected mice after exposure to a lethal dose of live Influenza A virus. Such a vaccine can be rapidly generated from any circulating virus making it relevant to any prevailing epidemic. The mRNA for the vaccine can be synthesized in a single step and does not require any additional steps other than purification.

## Methods

### Materials

Dulbecco’s Modified Eagle Medium (DMEM), Opti-MEM, 0.05% trypsin/0.53 mM EDTA, OptiPRO^TM^ SFM medium and GlutaMAX^TM^-I were all purchased from Gibco (Grand Island, NY). Fetal bovine serum (FBS) was purchased from Atlanta Biologicals (Lawrenceville, GA). Gentamicin Sulfate was from Corning (Corning, NY). All restriction enzymes, DNA polymerase I (Large (Klenow) Fragment), T4 DNA ligase, Deoxynucleotides, Anti-Reverse Cap Analog (ARCA), Antarctic Phosphatase, High-Efficiency Competent *E. Coli* Cells [NEB 10-beta] and HiScribe™ T7 Quick High Yield RNA Synthesis Kits were from New England BioLabs (Ipswich, MA). Hi-Lo DNA Markers were obtained from Minnesota Molecular, Inc. (Minneapolis, MN). QIAprep Spin Miniprep Kit was from Qiagen (Germantown, MD). LB Broth was purchased from Alfa Aesar (Ward Hill, MA). TransIT-mRNA lipid transfection kit was from Mirus Bio (Madison, WI). CellTiter 96® Aqueous One Solution Cell Proliferation Assay was from Promega (Madison, WI). Ethidium homodimer was purchased from Molecular Probes (Eugene, OR). Linear 25 kDa polyethylenimine (PEI) was obtained from Polysciences (Warrington, PA). Type II collagenase was from Worthington Biochemicals (Lakewood, NJ). Horse serum, laminin, and F(ab’)2 Goat anti-Rabbit IgG (H + L) Alexa FluorTM Plus 488 antibody and F(ab’)2 Goat anti-Mouse IgG (H + L) Alexa Fluor Plus 488 (cross-adsorbed) antibody were purchased from Invitrogen (Waltham, MA). Chick embryo extract was from US Biological (Swampscott, MA) and matrigel was from BD Biosciences (Franklin Lakes, NJ). Polyplus in vivo-jetRNA + ® Transfection Reagent was from Genesee Scientific (El Cajon, CA). Influenza A H1N1 (A/Puerto Rico/8/1934) Hemagglutinin / HA Protein (ECD, His Tag) and Rabbit Influenza A H1N1 Hemagglutinin (HA) monoclonal antibody were purchased from Sino Biological (Beijing, China). The Influenza A/PR/8/34 virus was generated by reverse genetics as described [21].

### Vectors

All DNA plasmids used for transfection (expression plasmids) or in vitro mRNA synthesis (template plasmids) contained a prokaryotic origin of replication and an ampicillin resistance gene for selection and were amplified in *E. coli* cells (NEB 10-beta, New England BioLabs, NEB# C3019H) in LB Broth (Alfa Aesar, Cat. No.H26676) at 37 ^o^C overnight. The plasmids were then isolated from *E. coli* and purified using QIAprep Spin Miniprep Kit (Qiagen, Cat. No. 27106) following the manufacturer’s protocols.

Expression DNA Plasmids I and II were designed for intracellular transcription of mRNAs that encoded eGFP and did not have any IRES sequences (see Fig. 2 for all plasmid schematics). Expression DNA Plasmids III and IV transcribed mRNAs encoding eGFP and did contain the EMCV IRES for eGFP expression. DNA Plasmids II and IV transcribed mRNA with self-folding double hairpins. DNA Plasmids I and III had a weakly structured 5’ UTR of the same length as the 5′ UTR in Plasmids II and IV (see Supplemental Data showing the predicted translation initiation and cleavage sites). Expression DNA Plasmids V–VIII were modifications of DNA Plasmids I-IV and were made by adding the mCherry ORF and a short 20 base pair (bp) linker upstream of eGFP in Plasmids I and II or upstream of the IRES in Plasmids III and IV. Expression Plasmid V transcribed mRNA with a weakly structured 5′ terminus and the mCherry ORF followed by a not-in-frame eGFP ORF without an IRES (designed to maintain a certain mRNA length and sequence, but which only allowed expression of mCherry). Expression Plasmid VI was similar to Plasmid V, but transcribed mRNA with a double hairpin on the 5′ terminus. Plasmid VII represents the classic bicistronic configuration that is commonly used to express two separate genes. In this configuration, mCherry expression was initiated by a cap-dependent mechanism and eGFP expression was initiated from an IRES. Plasmid VIII was similar to Plasmid VII, but transcribed mRNA that contained a double hairpin on the 5′ end.

All template DNA plasmids for in vitro mRNA synthesis were linearized by the BspQI (New England BioLabs, NEB# R0712S) restriction enzyme to define a 3′ end of synthesized mRNA and used as templates for T7 promoter-driven in vitro RNA synthesis. HiScribe™ T7 Quick High Yield RNA Synthesis Kits (New England BioLabs, NEB# E2050 and NEB#E2040) were used to generate up to 180 μg of RNA per reaction from 1 μg of each linearized DNA template following the manufacturer instructions; this was followed by DNase treatment to remove the DNA template and LiCl Precipitation. The synthesized RNAs were used as experimental mRNA vectors.

**Fig. 1:**
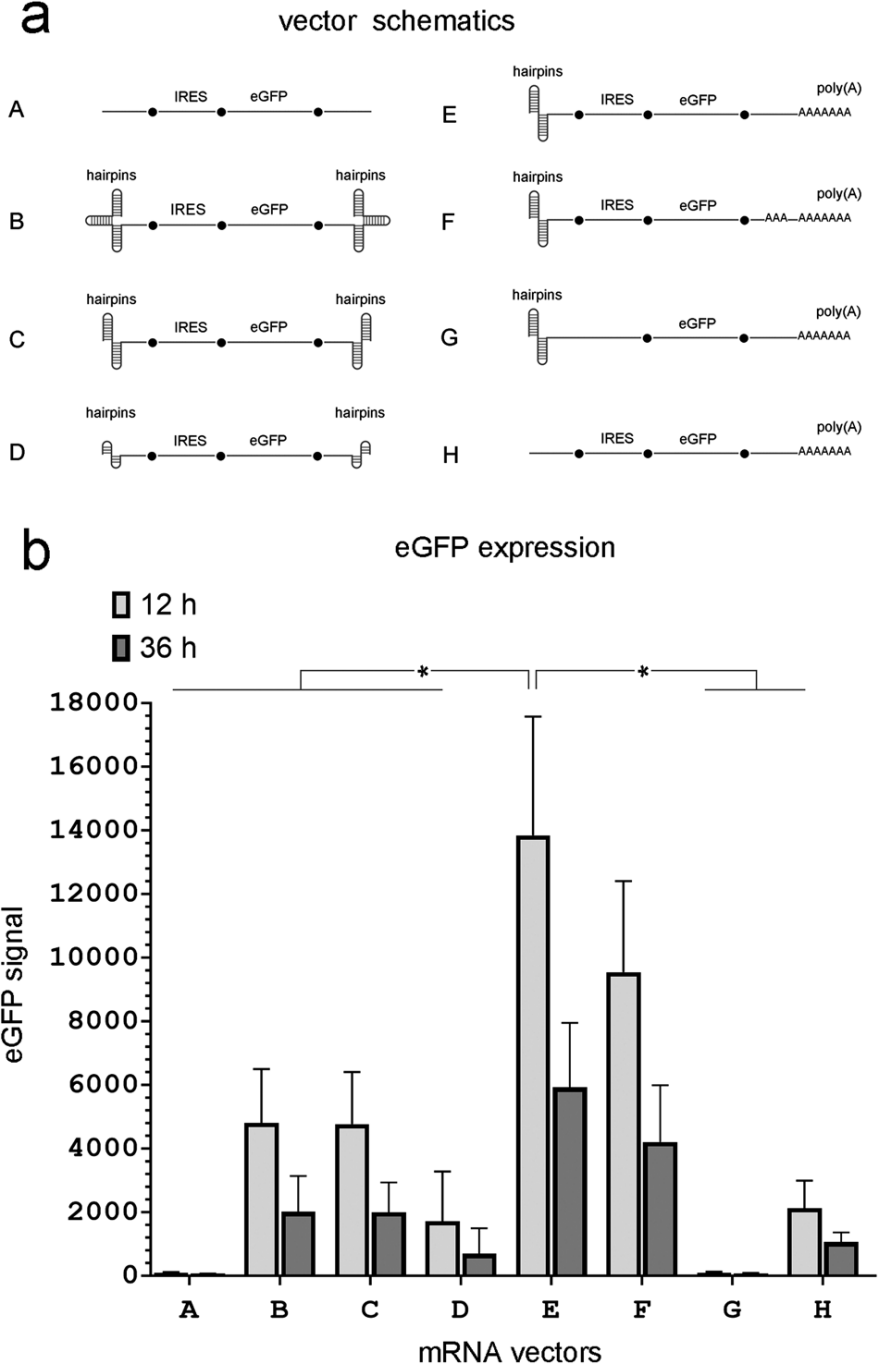
Capless mRNA vectors with double hairpins at the 5’ end demonstrated efficient IRES-initiated eGFP expression in MDCK cells. **a** mRNA vector schematics (see the text for structural details). **b** eGFP expression in MDCK cells 12 and 36 hours after transfection with equimolar amounts of the indicated mRNA vectors. (*n* = 4 independent experiments, *P < 0.05 shown for 12 hours only).

Some mRNAs were designed to form a terminal hairpin on only the 5’, whereas others formed terminal hairpins on both ends (see schematics for each experiment in figures and description). Terminal hairpins were either a double or triple hairpin and were comprised of two or three separate individual hairpins immediately adjacent to each other with no unpaired nucleotides in between (see supplemented data for sequences). All individual double-stranded hairpin stems had high G/C content. For most of the double hairpins at the 5′ end, each hairpin was 30 nucleotide (nt) long (a 13 bp stem and a 4 nt unpaired loop) and each of the double hairpins at the 3′ end was 34 nt (a 15 bp stem and a 4 nt unpaired loop). Therefore, the total length of these 5′ double hairpins was 60 nt and that of the 3′ double hairpins was 68 nt. (Of note: other stable terminal hairpin designs of a similar length (not presented here) showed similar effect on mRNA function.) One mRNA vector (Vector D, Fig. 1) had double hairpins at both ends which were comprised of individual hairpins with 16 nt (a 6 bp stem with a 4 nt unpaired loop). The stem of each individual hairpin was structurally different from the other individual hairpins to avoid potential interference due to the formation of secondary structures. All individual hairpins in the mRNA transcripts contained a single-stranded 4 nt A/U loop. When a poly(A) tail was included at the 3’ end of transcripts, it was comprised of 74 nt. In one mRNA transcript, we added a 30 nt internal poly(A) segment just upstream of the terminal 74 nt poly(A) tail which was connected to the terminal poly(A) tail with a 10 nt linker (i.e., a split or segmented poly(A) configuration). All poly(A) sequences were directly encoded in the DNA template used for their synthesis. Depending on the experiment, mRNA vectors were designed to support translation of eGFP, mCherry, and eGFP, or HA. For HA, the insert was amplified from Influenza A/PR/8/34 virus genomic RNA by RT-PCR and cloned into the DNA plasmid template using standard cloning methods (see supplemental data for details). Most of the mRNA vectors had an EMCV IRES sequence upstream of the eGFP or HA ORF to initiate translation. One experimental HA expression mRNA (also used as an experimental vaccine) was additionally treated with Antarctic Phosphatase (New England Biolabs, Cat. No. M0289S) to dephosphorylate the 5′ end. Two mCherry and eGFP expression mRNA vectors (mRNA 1C and 2C in Fig. 3) were capped with an Anti-Reverse Cap Analog (ARCA, New England Biolabs, Cat. No. S1411S) and were compared to their uncapped variants. Appropriate schematics of each vector are included with each figure.

### MDCK cells

MDCK cells (Madin-Darby Canine Kidney cell line [London line, FR-58]) were used in these experiments due to their reliable expression of HA and the reporter genes eGFP and mCherry when transfected with mRNA vectors that encode these proteins. MDCK cells were obtained from the International Reagent Resource, Influenza division, WHO collaborating center for surveillance, Epidemiology and control of influenza, Center for Disease Control and Prevention. MDCK cells were gradually adapted to grow in serum-free culture conditions as described [21]. MDCK cells were cultured in OptiPRO^TM^ SFM medium supplemented with 1 x GlutaMAX^TM^-I and 50 µg/ml Gentamicin at 37 °C in 5% CO_2_ and routinely passaged after reaching 80% confluency. Cells were harvested by 0.05% trypsin/0.53 mM EDTA digestion and counted with Coulter Z1 (Coulter Electronics). All cell cultures were routinely tested for mycoplasma contamination.

### Primary culture of mouse skeletal muscle

Skeletal muscle was excised from the lower limbs of 5-week-old BALB/c mice and digested in 0.2% type II collagenase as described elsewhere [22]. Mouse Skeletal Muscle Cells were collected via a 5 min centrifugation at 1800 *g* and purified from fibroblasts on uncoated culture dishes overnight at 37 °C in the culture medium containing 10% horse serum, 20% FBC, 1% chick embryo extract, and 50 µg/ml Gentamycin in DMEM. The unattached cells were centrifuged at 1800 *g* for 5 min, suspended in culture medium, plated onto dishes coated with matrigel, and incubated at 37 ^o^C overnight. The attached cells were trypsinized, suspended in culture medium and plated at 10^5^ cells/ml on 0.25% laminin-coated tissue culture dishes. After 5 days in culture, the culture medium was replaced with differentiation medium containing 2% horse serum, 1 x insulin-transferrin-selenium, and 50 µg/ml Gentamycin. Five days later most myoblasts differentiated into myocytes, which migrated, adhered and fused with one another to form small nascent myotubes with few nuclei (MC, myocytes/myotubes mix). The dishes with differentiated cells (MC) were directly used for transfection and analysis. All cell cultures were routinely tested for mycoplasma contamination.

### mRNA and plasmid DNA transfection and analysis

For mRNA transfection, MDCK (80% cell confluency) and MC (100% cell confluency) cells in a six-well plate dish were transfected with about 2 micrograms of mRNA using TransIT-mRNA lipid transfection kit (Mirus, Prod. No. 22024790). The exact amount of mRNA was adjusted in each experimental set to deliver its equimolar quantity. Cell viability was assessed by CellTiter 96® Aqueous One Solution Cell Proliferation Assay (Promega, G3580), and ethidium homodimer uptake (Molecular Probes, L-3224). For mCherry and eGFP expression analysis in MDCK cells, cells were trypsinized 12–36 h after transfection and analyzed by BD Biosciences Canto II cell analyzer in the University of South Alabama Flow Cytometry Core. For HA expression analysis membrane protein fractions from MDCK and MC cells were isolated and probed in 96 well plates with Rabbit Influenza A H1N1 Hemagglutinin (HA) monoclonal antibody (Sino Biological, Cat. No. 11684-R107) followed by F(ab’)2 Goat anti-Rabbit IgG (H + L) Alexa FluorTM Plus 488 antibody (Invitrogen, Cat. N. A48282) and analyzed on Synergy Neo 2 multi-mode reader (BioTek Instruments) to read a correspondent fluorescent signal.

**Fig. 2:**
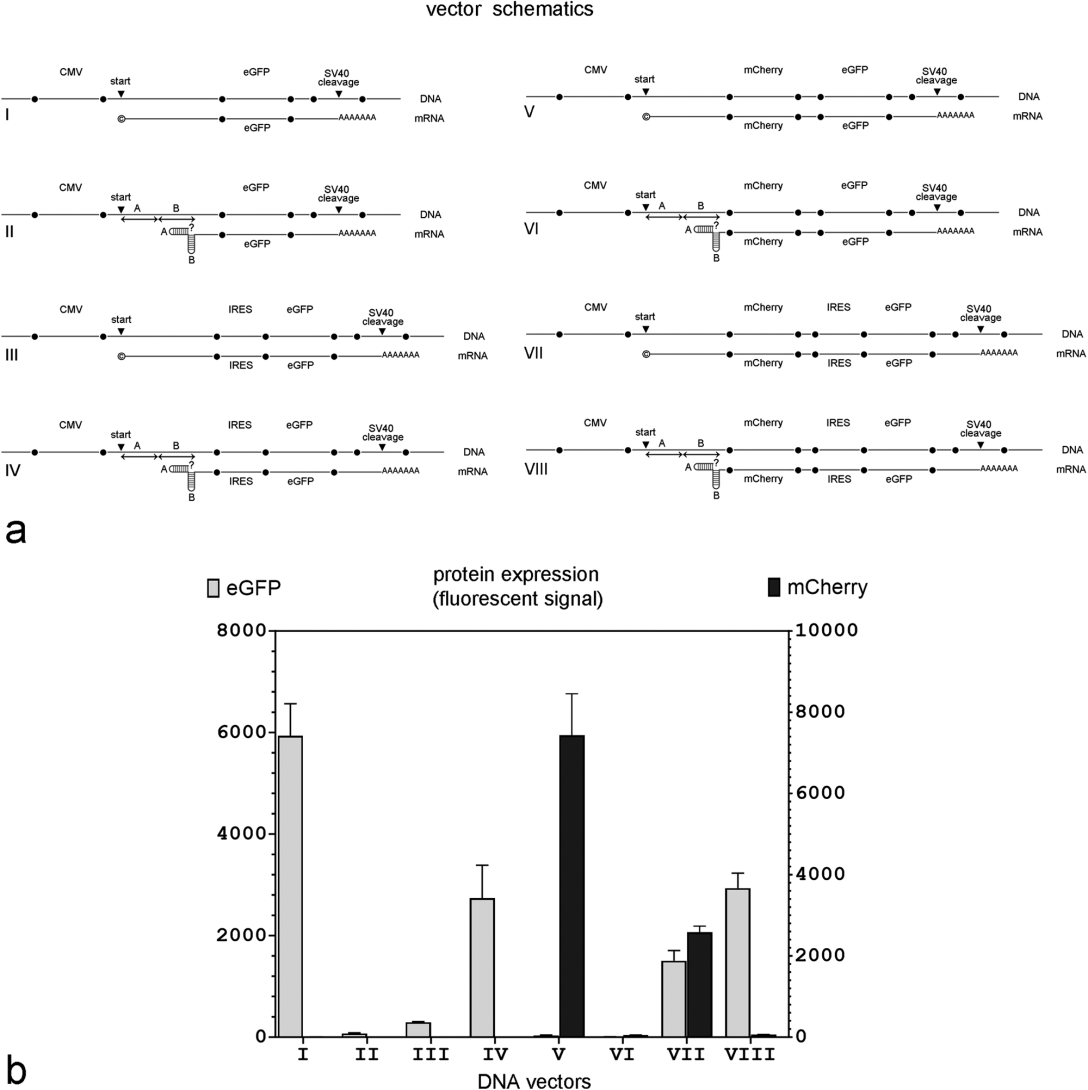
DNA expression plasmids that transcribe mRNAs with self-folding double hairpins at the 5’ end cannot initiate translation in the absence of an IRES. **a** Vector schematics; relevant parts of the DNA plasmids and their projected mRNA transcripts are shown together. **©** in mRNA transcripts I, III, V, and VII indicates that the transcript has likely been capped in vivo; **?** in Vectors II, IV, VI, and VIII indicates that the capping status of the transcript is unknown because it is not clear if mRNA with double hairpins in the 5’ end can be capped in vivo; ‘start’ - the transcription start site; ‘cleavage’ - the cleavage site within the SV40 polyadenylation signal; A and B - individual hairpin sequences folding into a double hairpin in mRNAs transcribed from DNA vectors II, IV, VI and VIII. **b** eGFP and mCherry expression in MDCK cells 48 hours after transfection with equimolar amounts of the indicated DNA plasmid. (*n* = 4 independent experiments).

For DNA plasmid transfection, all expression vectors (see description for Fig. 2) were complexed with PEI in Opti-MEM and used for MDCK cell transfection (at a 3:1 ratio of PEI to DNA (w/w)). MDCK cells (60% cell confluency in a six-well plate dish) were transfected with 2 micrograms of expression DNA plasmid I or an equimolar amount of DNA plasmids II, III, and IV or 2 µg of expression DNA plasmid V or an equimolar amount of DNA plasmids VI, VII, and VIII and analyzed for eGFP or mCherry/eGFP expression 48 hours post-transfection by BD Biosciences Canto II cell analyzer in the University of South Alabama Flow Cytometry Core.

### Animals

All animal studies were conducted under protocols approved by the animal care committee at the University of South Alabama.

Specific pathogen-free BALB/c(H-2d) mice were purchased from Charles River Laboratories (Kingston, NY). For mouse immunization, 1.5 µg of mRNA (either experimental or control) were complexed with pre-formed lipid-based nanoparticles and delivered to mice using an in vivo-jetRNA+ delivery system. mRNA for the experimental vaccine encoded the full-length HA of the Influenza A/PR/8/34 strain. This mRNA had a double hairpin at the 5’ end, an EMCV IRES to initiate HA translation, the HA ORF, and a poly(A) tail (Fig. 5b). The control mRNA had the same structure but with the eGFP ORF instead. Three immunizations were performed per animal on days 0, 10, and 21. The anti-HA antibody titers were calculated in serially diluted sera samples collected from each mouse 7 days before and 42 days after the first immunization by ELISA using a Synergy Neo 2 multi-mode reader (BioTek Instruments) to detect a fluorescent signal. Influenza A H1N1 (A/Puerto Rico/8/1934) Hemagglutinin / HA Protein (ECD, His Tag) (Sino Biological, Cat #: 11684-V08H) was used as the antigen and F(ab’)2 Goat anti-Mouse IgG (H + L) Alexa Fluor Plus 488 (cross-adsorbed) (Invitrogen, Cat. N. A48286) was used as the secondary antibody for signal detection (ELISA).

For the challenge experiments, 30 days after the last immunization with the experimental (HA) or the control (eGFP) mRNA vaccine, mice were challenged with a dose of live Influenza A/PR/8/34 virus that was 50 times its LD_50_ (1 × LD_50_ = 95 PFU/mouse) [21], by intranasal insufflation of 50 µL inoculum diluted in PBS. Mice were then monitored for weight loss, distress, and survival.

### Statistical analysis

Data are expressed as mean ± SD. Changes in protein expression, were compared using one-way ANOVA combined with Fisher post hoc analysis, with a *P* value < 0.05 considered significant. The desired level of statistical power was set at 0.80. In all figures “*n*“ represents the number of independent experiments. Each independent experiment was done on a separate day and was done in triplicate.

## Results

### Functionality of capless IRES-containing mRNA vectors with double hairpins at the 5’ end

Previously, we demonstrated that an mRNA vector that lacked a 5’ cap expressed eGFP more efficiently than a canonical (capped) mRNA vector as long as it contained an IRES in the 5’ UTR to initiate translation and triple hairpins at both ends to protect it from exonucleases [20]. To simplify the mRNA vector design, we tested the vector’s expression efficiency if it contained a double, instead of a triple, hairpin at the 5’ end. Schematics of the experimental and control vectors are shown in Fig. 1. In the experimental mRNA vectors the encephalomyocarditis virus (EMCV) IRES was included in the 5’ UTR upstream of the ORF for eGFP to drive its expression. In all experimental vectors, hairpins were included at the 5’ end, whereas the 3’ end differed between vectors and contained either a hairpin or poly(A) as shown in Fig. 1. Vectors that did not have hairpins at the 5’ end or did not contain an IRES for eGFP expression were included as controls.

As expected, the control mRNA vector without hairpins and a poly(A) tail failed to express eGFP even though it contained an IRES (Vector A, Fig. 1). The addition of triple hairpins at both ends (Vector B), rescued eGFP expression. These results are consistent with our previous findings [20]. Using two, instead of three, hairpins at each end, (Vector C) gave equivalent eGFP expression. Each of the double hairpins at the 5’ end was 30 nt (a 13 bp stem with a 4 nt loop) and each of the double hairpins at the 3’ end was 34 nt (a 15 bp stem with a 4 nt loop). If these individual hairpins were shortened to just 16 nt at both ends (a 6 bp stem with a 4 nt loop) eGFP expression was significantly decreased (Vector D). Based on these results, we chose to use the longer (30 nt) hairpins at the 5’ end to study the effect of modifying the 3’ end on vector performance.

Replacing the 3’ double hairpin with a terminal 74 nt poly(A) tail significantly improved eGFP expression (Fig. 1, Vector E). Including a 30 nt poly(A) fragment 10 nt upstream from the terminal 74 nt poly(A) tail (a design used in some mRNA vectors to improve DNA template stability [23]) decreased eGFP expression slightly in MDCK cells (Fig. 1, Vector F). Removing the IRES (Fig. 1, Vector G) or removing the 5’ double hairpins (Fig. 1, Vector H) significantly reduced eGFP expression indicating that these are required components of a functional capless mRNA vector.

### An IRES is required for protein expression from both endogenous and exogenous mRNAs harboring 5’ hairpins

The 5’ cap on mRNA is required for canonical translation. When mRNA is transcribed in vivo, it is capped at the 5’ end through the sequential actions of three enzymes, RNA triphosphatase, RNA guanylyltransferase, and guanine-N7 methyltransferase [24]. This is true whether the mRNA is transcribed from the host cell’s own DNA or whether it is transcribed from a DNA plasmid transfected into the cell. To determine whether the 5’ double hairpins would interfere with cap-dependent translation if mRNA was transcribed *endogenously* from a transfected DNA plasmid, we designed several DNA plasmid vectors for intracellular mRNA transcription and tested them in MDCK cells. All DNA plasmid vectors used for in vivo transcription were driven by a CMV promoter. Some of these DNA plasmids were designed to transcribe mRNAs with an unstructured 5’ end, whereas others were designed to transcribe mRNA that self-folded into a double hairpin at the 5’ end. The sequence of the 5’ double hairpin, where applicable, was the same as those in the in vitro synthesized mRNAs with the larger double hairpins described above (Fig. 1a, Vectors C and G). Schematics of the relevant parts of the DNA plasmids and their projected mRNA transcripts are shown in Fig. 2a. All DNA plasmid templates had the same transcription start site for the initiation of mRNA synthesis and the same SV40 cleavage site for polyadenylation (see Methods for details). The length of the 5’ UTR is predicted to be the same in all mRNA transcripts. Of note, the expectation is that all the mRNA transcribed from these DNA plasmids endogenously, will have a 5’ cap.

Figure 2b shows eGFP expression in MDCK cells 48 h post-transfection with equimolar amounts of each DNA expression plasmid. As expected, eGFP expression was robust in the control DNA Plasmid I. Adding a double hairpin to the 5’ end of the projected transcript (Plasmid II) prevented eGFP expression but adding an IRES upstream of the GFP ORF (Plasmid IV) rescued eGFP expression to about half maximum. Interestingly, eGFP expression was minimal from expression Plasmid III which generated an mRNA transcript that contained *both* a 5’ cap and an IRES but without 5’ hairpins. This suggested that there was some translation initiation conflict between the 5’ cap and the IRES that was prevented by the hairpins.

To directly compare cap-dependent and IRES-dependent protein expression from the same mRNA transcript, we modified all four DNA plasmids by adding the mCherry ORF and a short 20 bp linker upstream of the eGFP ORF in expression Plasmids I and II (to make Plasmids V and VI) or upstream of the IRES in expression Plasmids III and IV (to make Plasmids VII or VIII). In these new plasmids, mCherry expression required a functional cap, whereas eGFP expression required a functional IRES.

As shown in Fig. 2b, the first gene, mCherry, was only expressed when the transcribed mRNA did not have a 5’ double hairpin (Plasmids V and VII); in contrast, the second gene, eGFP, was expressed regardless of whether the 5’ end contained double hairpins or a cap as long as a functional IRES was present immediately upstream (Plasmids VII and VIII). This confirmed that the failure to express protein from plasmids that contained 5’ hairpins but lacked an upstream IRES (Plasmids II (eGFP), VI (mCherry), and VIII (mCherry)) was not due to transcript degradation, but rather a failure of translation.

**Fig. 3:**
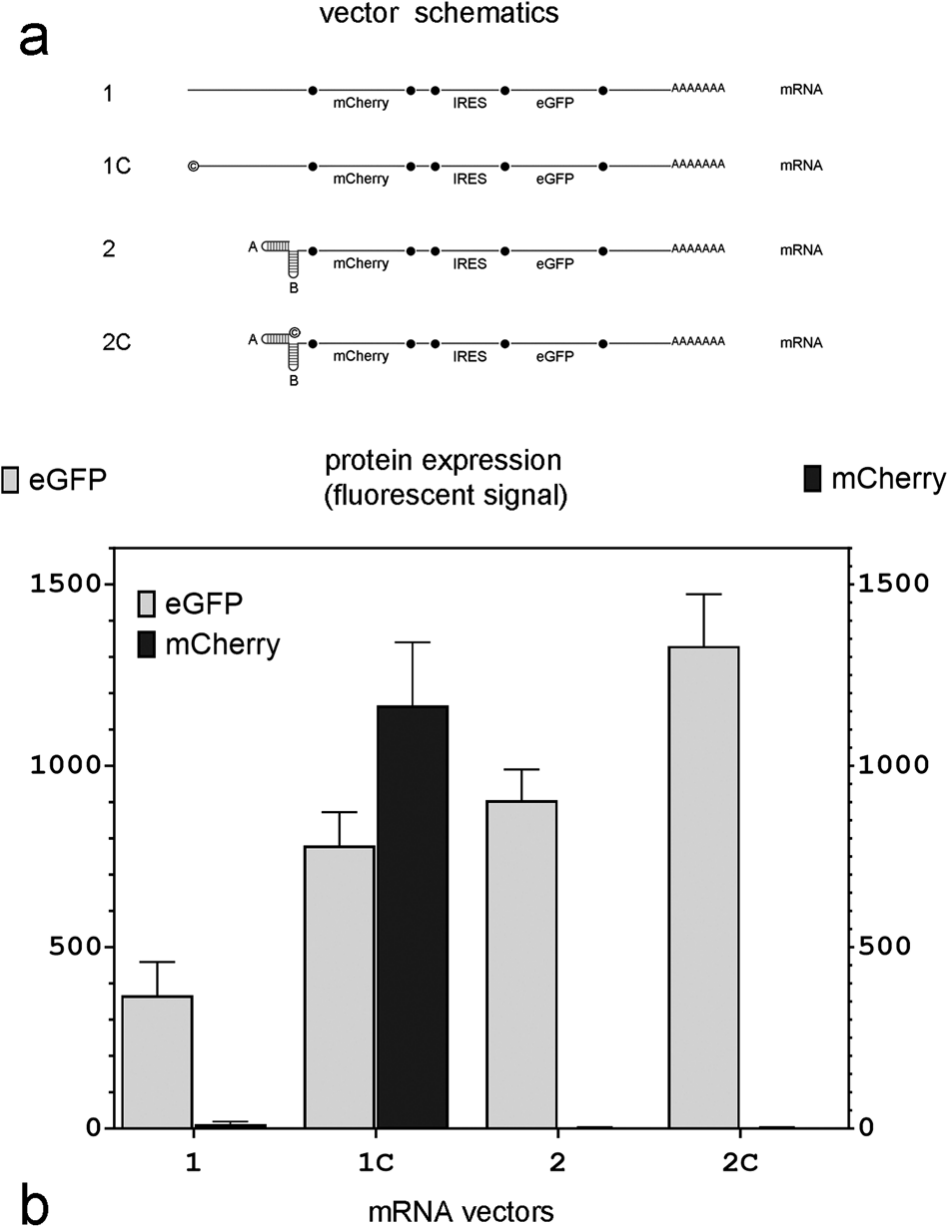
Double hairpins at the 5’ end interfere with cap-dependent translation of mCherry. **a** mRNA vector schematics; © in vectors 1C and 2C indicates that the transcript has been capped using an Anti-Reverse Cap Analog (ARCA); A and B: individual hairpin sequences that comprise the double hairpin in mRNA vectors 2 and 2C. **b** eGFP and mCherry expression in MDCK cells 12 h after mRNA transfection with equimolar amounts of each vector (*n* = 4 independent experiments).

What was not clear was whether the failure to initiate translation from the 5’ end of mRNA was due to a failure to cap a transcript that had likely already folded into a hairpin, or whether it was due to an inability of the 5’ cap located at the base of the hairpin to bind to the ribosome to initiate translation. To clarify if double hairpins in the 5’ terminus can directly inhibit expression from capped mRNA, we designed two DNA templates for in vitro mRNA synthesis with similar sequences as the projected mRNAs transcribed in vivo from DNA expression Plasmids VII and VIII (Fig. 2). The only differences between the in vivo and in vitro transcribed mRNAs were the sequences at the end of the 3’UTR and the length of the poly(A) tail (74 nt for the in vitro synthesized mRNAs, probably longer in the in vivo synthesized mRNAs). Using ARCA when synthesizing mRNA Vectors 1C and 2C (Fig. 3a) ensured that the transcript was capped regardless of its 5’ structure (i.e., regardless of whether it had double hairpins or not). We then compared eGFP and mCherry expression in MDCK cells 12 h after they were transfected with equimolar concentrations of mRNA.

Cells transfected with uncapped mRNA lacking 5’ double hairpins (Fig. 3; mRNA 1) did not express mCherry, whereas cells transfected with similar mRNA which was capped (Fig. 3, mRNA 1C) did. However, if the transcribed mRNA had a double hairpin at the 5’ terminus, mCherry was not expressed regardless of whether it was capped (Fig. 3, mRNA 2C) or not (Fig. 3, mRNA 2). This indicated that the presence of the 5’ double hairpin directly interfered with the cap-dependent translation of mCherry. This explains the absence of eGFP expression in cells transfected with DNA expression Plasmid II and mCherry expression in cells transfected with DNA expression Plasmids VI and VIII in Fig. 2 and mRNA transcript 2C in Fig. 3. In contrast, eGFP, whose translation was initiated from an IRES in all the in vitro synthesized transcripts shown in Fig. 3, was expressed from all four mRNAs, although in varying levels.

### Effect of polyadenylation and dephosphorylation of capless mRNA vectors on HA expression in vitro and anti-HA antibody induction in vivo

We next tested if a capless mRNA protected by a double hairpin at the 5’ end could express a membrane antigen at sufficiently high levels to be used as a vaccine. The antigen we chose was hemagglutinin (HA), the Influenza A surface protein responsible for virus binding to epithelial cell surface receptors [25] whose expression, endoplasmic reticulum membrane translocation, and proper folding includes additional steps beyond that required for eGFP translation [26]. In several of the mRNA vectors described in Fig. 1, we replaced eGFP with the full-length Influenza A HA. Because we planned to test the best-performing mRNA vector as an intramuscular vaccine in BALB/c mice, we transfected the mRNA vectors into myocytes and partially merged myotubes (MC) isolated from BALB/c mice in addition to the MDCK cells used in the previous experiments.

**Fig. 4:**
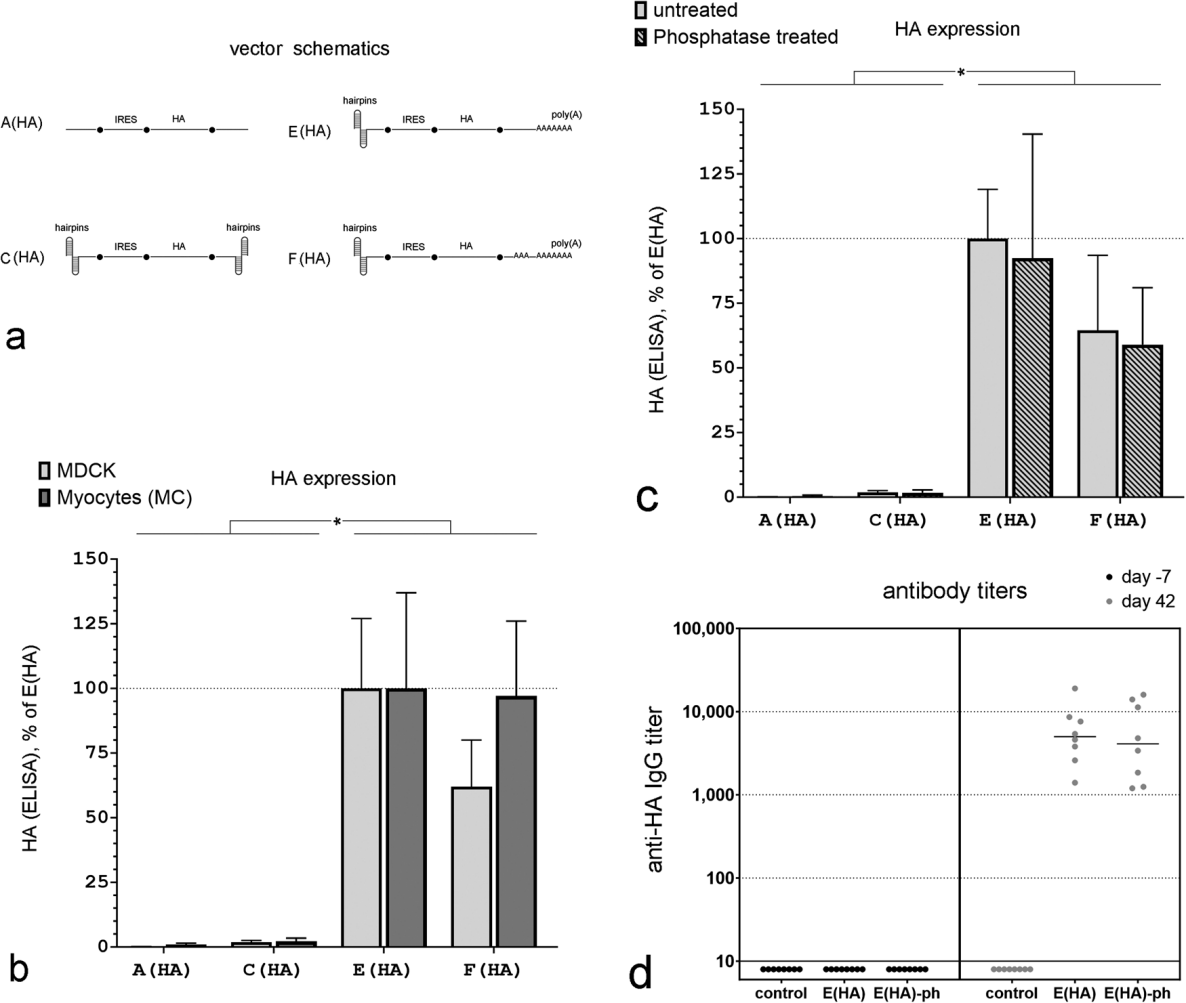
Capless IRES-initiated mRNA vectors with double hairpins at the 5’ end require a poly(A) tail for HA expression in MDCK cells and mouse myoblasts and induce anti-HA antibody production when delivered as a vaccine. **a** mRNA vector schematics. **b** HA expression in MDCK cells and mouse myoblasts (MB) 12 h after transfection with equimolar concentrations of the indicated mRNA vectors. ELISA data are normalized to the signal after transfection with vector E(HA) separately for MDCK cells and myocytes. (*n* = 4 independent experiments done at separate times, *P < 0.05). **c** HA expression in MDCK cells 12 h after transfection with equimolar concentrations of phosphatase-treated or -untreated mRNA vectors. ELISA data are normalized to the signal after transfection with the untreated mRNA vector E(HA). (*n* = 4 independent experiments done at separate times, *P < 0.05). **d** Anti-HA IgG titers three weeks after the last vaccination in individual mice immunized with a HA-mRNA vaccine that contained either the E(HA) or E(HA)-ph mRNA vectors. Control: data for non-immunized mice. For all comparisons, blood was collected 7 days before the first immunization and 21 days after the third immunization.

In contrast to our results with eGFP (Fig. 1), an mRNA vector with double hairpins on both ends (Fig. 4b, Vector C(HA)) expressed only a minimal amount of HA 12 h post-transfection in both MDCK cells and mouse myocytes (MC); its expression levels were barely above those detected in cells transfected with a vector with no hairpins at all (Fig. 4b, Vector A(AH)). However, replacing the 3’ hairpin with a 74 nt poly(A) tail markedly improved HA expression in both cell types (Fig. 4b, Vector E(HA)). Replacing the 3’ double terminal hairpin with a longer, segmented poly(A) tail (30 nt + 74 nt) did not improve HA expression over a vector with the single, shorter (74 nt) poly(A) configuration (Fig. 4b, Vectors E(HA) and F(HA)).

Capping of conventional mRNA synthesized in vitro may not be 100% efficient leaving a triphosphate at the 5’ end. This triphosphate can be recognized by RIG-I and leads to immune activation in cells [27]. Therefore, prior to use, some synthetic mRNAs are treated with phosphatases to remove the 5’ triphosphate to prevent this immunological response [27]. In capless mRNA, the 5’ end is buried and thus it is not clear whether the terminal triphosphate can trigger this immune response. To determine if dephosphorylation of the triphosphate on the 5’ end of the double-hairpin vector had any impact on protein expression in vitro, we treated all mRNAs with alkaline phosphatase prior to transfection (A(HA)-ph, C(HA)-ph, E(HA)-ph, F(HA)-ph). Dephosphorylation of A(AH), C(AH), E(HA), and F(AH) mRNA had no significant impact on HA expression in MDCK cells (Fig. 4c).

**Fig. 5:**
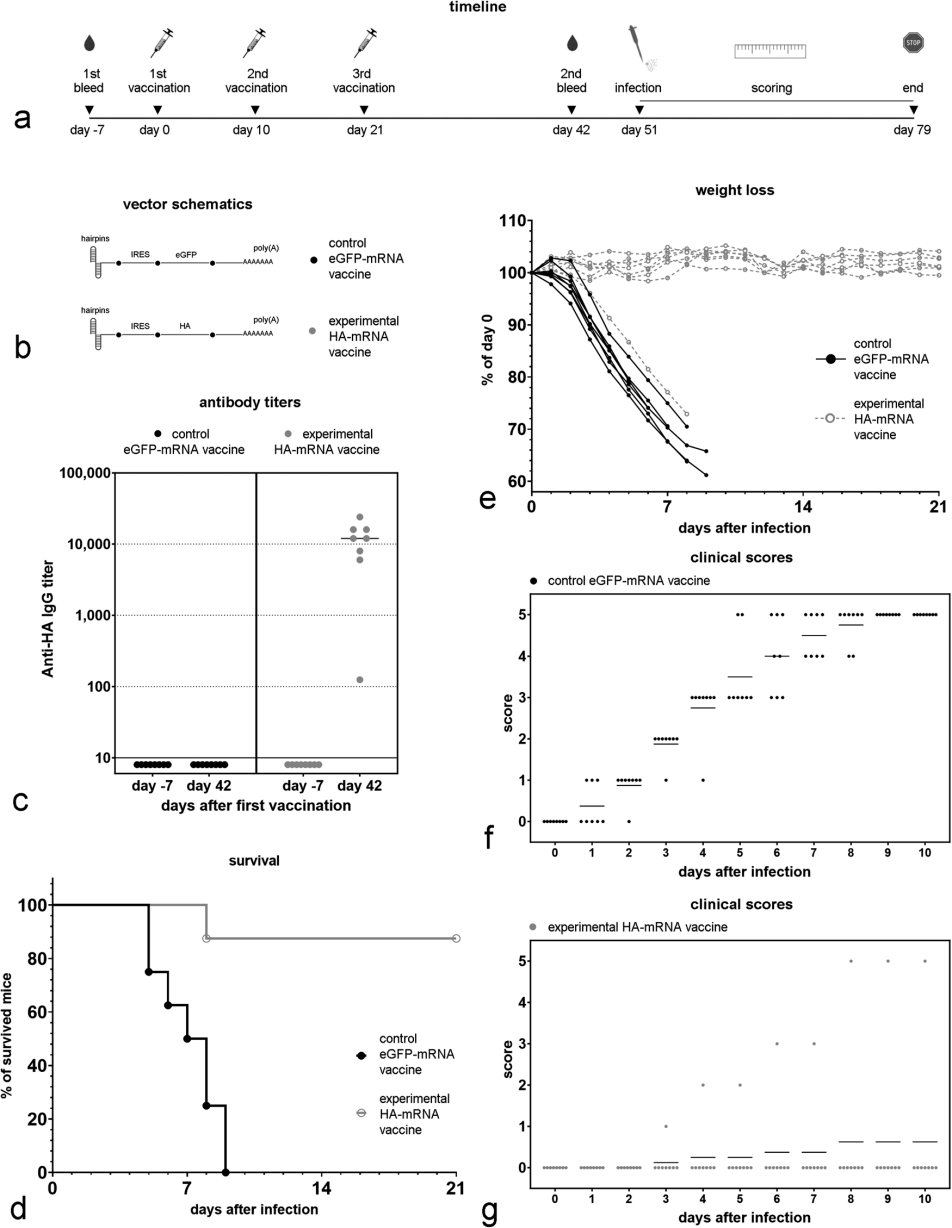
Immunization with a vaccine that includes a capless mRNA that has double hairpins at the 5’ end and encodes the EMCV IRES linked to the HA ORF protects mice against a lethal dose of Influenza A. **a** Timeline of the experiment. **b** Schematic of the mRNA vectors used in the vaccines. **c** Anti-HA antibody titer 7 days before the first vaccination and then 21 days after the last vaccination (42 days after the first vaccination). **d** Survival data for vaccinated groups for the three weeks after challenge with the influenza A virus; no animals died after 3 weeks. **e** Weight loss in vaccinated mice after Influenza A (A/PR/8/34) virus infection ((50 × LD_50_) (LD_50_ = 95 PFU/mouse) for the first three weeks after challenge. **f** Clinical scores assigned for eGFP-mRNA vaccinated mice for the first ten days after a live Influenza A virus challenge. **g** Clinical score assigned for HA-mRNA vaccinated mice for the first ten days after a live Influenza A virus challenge. The following clinical scores were assigned: 0 = normal, 1 = slightly ruffled, 2 = ruffled fur, 3 = ruffled fur and inactive, 4 = hunched/moribund, and 5 = dead.

We then tested the best-expressing in vitro mRNA capless vector, (E(HA)), in mice to determine whether it was able to induce an antibody response against HA. 1.5 µg of mRNA were complexed with pre-formed lipid-based nanoparticles and delivered to mice using an in vivo-jetRNA+ transfection reagent (HA-mRNA vaccine). Three immunizations were performed per animal on days 0, 10, and 21 (Fig. 5a). Antibodies against HA were measured 1 week prior to, and 42 days after, the initial immunization. Vaccination with this capless double hairpin HA-mRNA vaccine generated significant HA antibody titers in all mice. Removing the 5’ triphosphate on this vector using phosphatase (E(HA)-ph) had no significant impact on anti-HA titers in mice (Fig. 4d).

### Vaccination with a capless IRES-containing mRNA that expresses HA protects mice against a lethal dose of Influenza A

We then tested whether a capless mRNA vector encoding the full-length Influenza A surface protein hemagglutinin (HA) linked to an EMCV IRES and having a double hairpin structure on its 5’ end (Fig. 4) could protect BALB/c mice against Influenza A. Because the mRNA Vector E(HA) generated the highest anti-HA titer in the previous experiment, we selected it for use in these experiments (Fig. 5B); mRNA vector E, expressing eGFP, was used as a negative control. 1.5 µg of mRNA were complexed with pre-formed lipid-based nanoparticles and delivered to mice using an in vivo-jetRNA+ transfection reagent. Three immunizations were performed per animal on days 0, 10, and 21 (Fig. 5a). The mRNA vectors used in the vaccine formulation are shown in Fig. 5b. Anti-HA antibody titers were measured in sera collected from each mouse 7 days before and 42 days after the first immunization (Fig. 5c). Seven out of eight mice injected with the HA-mRNA vaccine, but none of the mice injected with the control eGFP-mRNA vaccine showed high anti-HA antibody titers 21 day after the last immunization (Fig. 5c).

We then exposed mice to 50 times the lethal dose of live Influenza A virus (strain A/PR/8/34). While all the mice immunized with the control eGFP-mRNA vaccine died, only one of the eight mice vaccinated with the capless mRNA vaccine encoding HA died (Fig. 5d); the sole vaccinated mouse that died was the one that did not generate a high anti-HA titer following vaccination (Fig. 5c). In addition, most of the HA-mRNA vaccinated mice had no weight loss (Fig. 5e) and demonstrated minimal clinical impact after challenge (Fig. 5f, g).

## Discussion

In vivo, capping of the 5’ end of mRNA occurs enzymatically in the nucleus after the first 20 to 30 nucleotides are incorporated into the RNA polymerase II generated transcript [28, 29]. This 5′ cap has multiple functions including transcript splicing, nuclear export of the mature mRNA to the cytoplasm, initiation of translation, and protection of the 5’ end from exonuclease cleavage [9]. Currently, most mRNA synthesized in vitro for use as vaccines or therapeutics also have a 5’ cap but its role is mostly limited to initiating translation and protecting against exonucleases since there is no need for splicing or nuclear export of the transcript, unlike mRNA that is synthesized and processed by RNA polymerase II in vivo [30]. However, capping contributes significantly to the cost of producing mRNA in vitro.

Previously, we described a linear capless and tailless mRNA in which both ends were sealed with triple hairpins and included an IRES in the 5’ UTR to initiate protein translation [20]. We show herein that a double hairpin is as good as a triple hairpin in stabilizing the mRNA vector, if the hairpins are of a certain length and stability. The switch to double hairpins makes the vectors shorter and simpler without any loss of protein expression and should decrease the theoretical risk of steric interference of the hairpin with the rest of the transcript. In addition, unlike the previous mRNA vector that contained hairpins at the 3’ end, this vector was re-designed to include a 3’ poly(A) tail instead of a hairpin. This was necessary because while the intracellular reporter gene eGFP could be expressed from tailless vectors, HA was not efficiently expressed in capless vectors that lacked a 3’ poly(A) tail. We think this is due to the differences in processing between cytosolic and membrane or secreted proteins. The lack of a poly(A) tail may result in decreased expression of certain membrane and secreted proteins whose translation is believed to be poly(A) dependent [31]. Transcripts which encode these types of proteins are targeted to, and translated on, the endoplasmic reticulum (ER). Although the targeting of these transcripts to the surface of the ER can be mediated by a signal sequence, it is becoming increasingly clear that additional poly(A) dependent ER-localization and regulation pathways exist [31, 32]. mRNA can bind to ER due to the interaction of the poly(A) tail with different membrane receptors that can affect ER protein expression [33, 34].

When complexed with pre-formed lipid-based nanoparticles and injected into mice, this capless mRNA vector generated high titers of anti-HA IgG antibodies and all but one of the mice survived when exposed to 50 times the LD_50_ of Influenza A virus. In contrast, mice vaccinated with capless mRNA vectors encoding GFP developed no anti-HA antibodies and all succumbed following live viral infection. Other investigators have previously demonstrated that canonical (capped) mRNA vectors encoding HA generate antibodies that are protective against infection with live influenza A virus [35, 36]. This is the first report that a linear capless mRNA encoding HA is also protective.

As part of the work to develop this capless mRNA vector, we observed that a terminal double hairpin on the 5’ end completely prevented translation from capped mRNAs produced in vitro. Interestingly, the presence of 5’ terminal double hairpins in mRNAs transcribed in vivo by Pol II polymerase (after transfection of host cells with DNA plasmids designed to transcribe such mRNAs from a CMV promoter), also inhibited cap-dependent protein translation. It is not clear if the hairpins prevent cap-dependent translation by physically blocking the cap from binding to the ribosomes or through some other mechanism, but it suggests that terminal double hairpins at the 5’ end of mRNA are not conducive to cap-dependent translation at least not in the configuration described here. If a terminal double hairpin is present at the 5’ end of an mRNA transcript, regardless of whether it was synthesized in vitro or in vivo, the transcript requires an internal translation initiation site to express protein. In the vectors described herein, the internal translation initiation site was an EMCV IRES, but it could include an IRES from other viruses or other internal translation initiation sites.

Unlike the 5’ cap, which does not initiate translation in a tissue/cell-specific way [11], there is evidence that internal translation initiation sites, such as an IRES, do express better in some cell types than in others [37, 38]. Therefore, one potential way to exploit this observation is to design DNA or viral vectors, including integrative vectors, that would ultimately transcribe mRNA with a terminal hairpin on the 5’ end in vivo and drive transcription from an IRES (or other internal translation initiation site) rather than from the cap. This might make it possible to create cell-specific capless mRNA vectors that are driven by IRES translation. Whether this is possible will require additional studies.

Synthetic circular RNAs (circRNA), in which the 5’ and 3’ ends are ligated together, is another form of RNA that can function without a 5’ cap as long as an IRES or other internal translation initiation site is present [39]. Because circRNAs do not have 5′ and 3′ ends, they are more stable than the linear uncapped and non-polyadenylated form of the same sequence [40]. One important difference between linear (capped and capless) mRNA and circRNA is the absence of a poly(A) tail in circRNA.The lack of a poly(A) tail may result in decreased expression of certain membrane and secreted proteins whose translation is more poly(A) dependent [31]. Although adding *internal* poly(A) or pAC spacer sequences can promote translation of *cytoplasmic* proteins like GFP and Gaussia luciferase by facilitating interactions with eukaryotic translation initiation factor eIF4G and polyA-binding protein (PABP) [40–42], such modifications may not be as effective as the terminal polyadenylation of a linear mRNA. This was true in our experiments where eGFP could be effectively expressed in cells by capless IRES-containing mRNA vectors that contained a hairpin at the 3’ end, whereas the membrane protein, HA, required a poly(A) tail for robust expression. Additional studies will be required to determine if this observation impacts the efficacy of different mRNA vectors.

In mRNA transcribed in vitro, the poly(A) tail is often encoded into the plasmid DNA template rather than added by poly(A) polymerase in a separate step. However, plasmid DNA that encodes long homopolymeric (i.e., poly(A)) stretches frequently recombines during bacterial amplification which can interfere with transcript function [43]. Segmentation of the poly(A) tail (i.e., including a short segment of non-adenosine nucleotides within the poly(A) tail) can reduce unwanted recombination of these poly(A) stretches in plasmid vectors [23]. In addition, because these segmented poly(A) tails can be longer than conventional poly(A) tails, the inclusion of an internal poly(A) sequence may lead to better mRNA expression. We tested whether a segmented poly(A) tail, similar to that found in the SARS-CoV-2 vaccine (World Health Organization MedNet document 11889) improved function in a capless mRNA vector. In the capless system described here, mRNA performance was not improved by including a segmented poly(A) tail (30 nt + 74 nt) compared to a shorter conventional one (74 nt). This may be explained by less reliance on the EMCV IRES on poly(A)-dependent translation initiation [44] than that of a conventional cap, but other factors may also be involved.

5’ triphosphates which are present in uncapped mRNA synthesized in vitro, can be recognized by RIG-I and lead to immune activation in cells [27, 45]. Since capping is not 100% efficient, mRNA synthesized in vitro is frequently treated with phosphatases prior to use as a vaccine or therapeutic to prevent this [46, 47]. Pre-treatment with a phosphatase to remove the 5’ triphosphates in the capless hairpin-containing mRNA vector described here, had no impact on HA expression in vitro or on the induction of anti-HA antibody titers in vivo. It is possible that the cell receptors that initiate the cell immune response did not recognize the terminal triphosphate due to physical limitations from the double hairpin, but we did not specifically test this. In our experiments, the capless vectors functioned similarly in vitro and in vivo regardless of whether they were treated with phosphatases.

These results demonstrate that exogenously synthesized mRNA that lacks a 5’ cap but contains an internal ribosome entry site (IRES) to initiate translation and hairpins at the terminal 5’ end to protect against exonucleases, can express the Influenza A surface protein hemagglutinin (HA) in sufficiently high levels to generate protective antibodies in mice. While previous studies have demonstrated the protective efficacy of a mRNA vaccine against Influenza A in mice [2, 35, 48–50], this is the first demonstration that a linear capless mRNA can also be protective.

## Supplementary information

Supplemental Data

## Data Availability

Data will be made available upon request.
