# Supplementary material for: A capless hairpin-protected mRNA vaccine encoding the full-length Influenza A hemagglutinin protects mice against a lethal Influenza A infection
Source: Gene Ther. 2025 Feb 23;32(4):349–58. doi: 10.1038/s41434-025-00521-0 (PMC12310517; doi:10.1038/s41434-025-00521-0)
Supplement: Supplementary file 1 — Supplemental Data [file 41434_2025_521_MOESM1_ESM.docx]

**Supplemental Data**

**5’ and 3’ triple hairpin sequences used in mRNA vector B:**

GGGCCGUCCGGGCAUUUGCCCGGACGGCCCCGUGCCUGCCUGCAUAUGCAGGCAGGCACGCCCGGCAGCCCGCAUUUGCGGGCUGCCGGG>>>>>>

>>>>>>GCGCGCCAGGGCGCCAAAUGGCGCCCUGGCGCGCCGACGGCUCGGGAGGAUAUCCTCCCGAGCCGUCGCCGCGGGUGGGCGCCAAAUGGCGCCCACCCGCGG

**5’ and 3’ double hairpin sequences used in mRNA vectors C and C(HA):**

GGGCCGUCCGGGCAUUUGCCCGGACGGCCCCCCGGCAGCCCGCAUUUGCGGGCUGCCGGG>>>>>>

>>>>>>GCGCGCCAGGGCGCCAAAUGGCGCCCUGGCGCGCCCGCGGGUGGGCGCCAAAUGGCGCCCACCCGCGG

**5’ and 3’ short double hairpin sequences used in mRNA vector D:**

GGGGCCAUUUGGCCCCGCCGGCAUUUGCCGGC>>>>>>

>>>>>>GCGCGCAUUUGCGCGCCCGCGGAUUUCCGCGG

**5’ double hairpin sequence used in mRNA vectors E, F, G, E(HA) and F(HA):**

GGGCCGUCCGGGCAUUUGCCCGGACGGCCCCCCGGCAGCCCGCAUUUGCGGGCUGCCGGG>>>>>>

**Sequence of CMV promoter/enhancer and its translation start site region used in DNA vectors I, III, V, VII. CMV promoter/enhancer sequence is highlighted in yellow. The predicted translation start site is indicated by a red arrow:**

>>>>>>GACATTGATTATTGACTAGTTATTAATAGTAATCAATTACGGGGTCATTAGTTCATAGCCCATATATGGAGTTCCGCGTTACATAACTTACGGTAAATGGCCCGCCTGGCTGACCGCCCAACGACCCCCGCCCATTGACGTCAATAATGACGTATGTTCCCATAGTAACGCCAATAGGGACTTTCCATTGACGTCAATGGGTGGAGTATTTACGGTAAACTGCCCACTTGGCAGTACATCAAGTGTATCATATGCCAAGTACGCCCCCTATTGACGTCAATGACGGTAAATGGCCCGCCTGGCATTATGCCCAGTACATGACCTTATGGGACTTTCCTACTTGGCAGTACATCTACGTATTAGTCATCGCTATTACCATGGTGATGCGGTTTTGGCAGTACATCAATGGGCGTGGATAGCGGTTTGACTCACGGGGATTTCCAAGTCTCCACCCCATTGACGTCAATGGGAGTTTGTTTTGGCACCAAAATCAACGGGACTTTCCAAAATGTCGTAACAACTCCGCCCCATTGACGCAAATGGGCGGTAGGCGTGTACGGTGGGAGGTCTATATAAGCAGAGCTCGTTTAGTGAACCꜜGTCAGATCGCCTGGAGACGCCATCCACGCTGTTTTGACCTCCATAGCTGGGCAACGTGCT>>>>>>

**Sequence of CMV promoter/enhancer and its translation start site region used in DNA vectors II, IV, VI, VIII. CMV promoter/enhancer sequence is highlighted in yellow. The predicted translation start site is indicated by a red arrow. The double hairpin sequence is highlighted in blue.**

>>>>>>GACATTGATTATTGACTAGTTATTAATAGTAATCAATTACGGGGTCATTAGTTCATAGCCCATATATGGAGTTCCGCGTTACATAACTTACGGTAAATGGCCCGCCTGGCTGACCGCCCAACGACCCCCGCCCATTGACGTCAATAATGACGTATGTTCCCATAGTAACGCCAATAGGGACTTTCCATTGACGTCAATGGGTGGAGTATTTACGGTAAACTGCCCACTTGGCAGTACATCAAGTGTATCATATGCCAAGTACGCCCCCTATTGACGTCAATGACGGTAAATGGCCCGCCTGGCATTATGCCCAGTACATGACCTTATGGGACTTTCCTACTTGGCAGTACATCTACGTATTAGTCATCGCTATTACCATGGTGATGCGGTTTTGGCAGTACATCAATGGGCGTGGATAGCGGTTTGACTCACGGGGATTTCCAAGTCTCCACCCCATTGACGTCAATGGGAGTTTGTTTTGGCACCAAAATCAACGGGACTTTCCAAAATGTCGTAACAACTCCGCCCCATTGACGCAAATGGGCGGTAGGCGTGTACGGTGGGAGGTCTATATAAGCAGAGCTCGTTTAGTGAACCꜜGGGCCGTCCGGGCATTTGCCCGGACGGCCCCCCGGCAGCCCGCATTTGCGGGCTGCCGGG>>>>>

**5’UTR sequence used in mRNA vectors 1 and 1C:**

GGGAAUAAACUAGUAUUCUUCUGGUCCCCACAGACUCAGAGAGAACCCGCCACCGGACGCGUGCCUGGAGACGCCAUCCACGCUGUUUUGACCUCCAUAGAAGACACCGACUCUAGCUAGAGGCUAGGAUCC>>>>>>

**5’UTR sequence used in mRNA vectors 2 and 2C. The double hairpin sequence is highlighted in blue):**

GGGCCGUCCGGGCAUUUGCCCGGACGGCCCCCCGGCAGCCCGCAUUUGCGGGCUGCCGGGACGCGUGCCUGGAGACGCCAUCCACGCUGUUUUGACCUCCAUAGAAGACACCGACUCUAGCUAGAGGCUAGGAUCC>>>>>>

**EMCV IRES (gray) and eGFP (green) sequences in all IRES dependent DNA eGFP expression vectors and all plasmid DNA template vectors used for synthesis of IRES dependent eGFP expressing mRNAs. The start and stop codons are underlined:**

CCCCTCTCCCTCCCCCCCCCCTAACGTTACTGGCCGAAGCCGCTTGGAATAAGGCCGGTGTGCGTTTGTCTATATGTTATTTTCCACCATATTGCCGTCTTTTGGCAATGTGAGGGCCCGGAAACCTGGCCCTGTCTTCTTGACGAGCATTCCTAGGGGTCTTTCCCCTCTCGCCAAAGGAATGCAAGGTCTGTTGAATGTCGTGAAGGAAGCAGTTCCTCTGGAAGCTTCTTGAAGACAAACAACGTCTGTAGCGACCCTTTGCAGGCAGCGGAACCCCCCACCTGGCGACAGGTGCCTCTGCGGCCAAAAGCCACGTGTATAAGATACACCTGCAAAGGCGGCACAACCCCAGTGCCACGTTGTGAGTTGGATAGTTGTGGAAAGAGTCAAATGGCTCTCCTCAAGCGTATTCAACAAGGGGCTGAAGGATGCCCAGAAGGTACCCCATTGTATGGGATCTGATCTGGGGCCTCGGTGCACATGCTTTACATGTGTTTAGTCGAGGTTAAAAAAACGTCTAGGCCCCCCGAACCACGGGGACGTGGTTTTCCTTTGAAAAACACGATGATAATATGGCCACAACCATGGTGAGCAAGGGCGAGGAGCTGTTCACCGGGGTGGTGCCCATCCTGGTCGAGCTGGACGGCGACGTAAACGGCCACAAGTTCAGCGTGTCCGGCGAGGGCGAGGGCGATGCCACCTACGGCAAGCTGACCCTGAAGTTCATCTGCACCACCGGCAAGCTGCCCGTGCCCTGGCCCACCCTCGTGACCACCCTGACCTACGGCGTGCAGTGCTTCAGCCGCTACCCCGACCACATGAAGCAGCACGACTTCTTCAAGTCCGCCATGCCCGAAGGCTACGTCCAGGAGCGCACCATCTTCTTCAAGGACGACGGCAACTACAAGACCCGCGCCGAGGTGAAGTTCGAGGGCGACACCCTGGTGAACCGCATCGAGCTGAAGGGCATCGACTTCAAGGAGGACGGCAACATCCTGGGGCACAAGCTGGAGTACAACTACAACAGCCACAACGTCTATATCATGGCCGACAAGCAGAAGAACGGCATCAAGGTGAACTTCAAGATCCGCCACAACATCGAGGACGGCAGCGTGCAGCTCGCCGACCACTACCAGCAGAACACCCCCATCGGCGACGGCCCCGTGCTGCTGCCCGACAACCACTACCTGAGCACCCAGTCCGCCCTGAGCAAAGACCCCAACGAGAAGCGCGATCACATGGTCCTGCTGGAGTTCGTGACCGCCGCCGGGATCACTCTCGGCATGGACGAGCTGTACAAGTAA

**eGFP (green) sequences in all IRES independent DNA eGFP expression vectors and all plasmid DNA template vectors used for synthesis of IRES independent eGFP expressing mRNAs. The start and stop codons are underlined:**

ATGGTGAGCAAGGGCGAGGAGCTGTTCACCGGGGTGGTGCCCATCCTGGTCGAGCTGGACGGCGACGTAAACGGCCACAAGTTCAGCGTGTCCGGCGAGGGCGAGGGCGATGCCACCTACGGCAAGCTGACCCTGAAGTTCATCTGCACCACCGGCAAGCTGCCCGTGCCCTGGCCCACCCTCGTGACCACCCTGACCTACGGCGTGCAGTGCTTCAGCCGCTACCCCGACCACATGAAGCAGCACGACTTCTTCAAGTCCGCCATGCCCGAAGGCTACGTCCAGGAGCGCACCATCTTCTTCAAGGACGACGGCAACTACAAGACCCGCGCCGAGGTGAAGTTCGAGGGCGACACCCTGGTGAACCGCATCGAGCTGAAGGGCATCGACTTCAAGGAGGACGGCAACATCCTGGGGCACAAGCTGGAGTACAACTACAACAGCCACAACGTCTATATCATGGCCGACAAGCAGAAGAACGGCATCAAGGTGAACTTCAAGATCCGCCACAACATCGAGGACGGCAGCGTGCAGCTCGCCGACCACTACCAGCAGAACACCCCCATCGGCGACGGCCCCGTGCTGCTGCCCGACAACCACTACCTGAGCACCCAGTCCGCCCTGAGCAAAGACCCCAACGAGAAGCGCGATCACATGGTCCTGCTGGAGTTCGTGACCGCCGCCGGGATCACTCTCGGCATGGACGAGCTGTACAAGTAA

**mCherry (red) sequences in all DNA mCherry expression vectors and all plasmid DNA template vectors used for synthesis of mCherry expressing mRNAs. The start and stop codons are underlined:**

ATGGTGAGCAAGGGCGAGGAGGACAACATGGCCATCATCAAGGAGTTCATGCGCTTCAAGGTGCACATGGAGGGCTCCGTGAACGGCCACGAGTTCGAGATCGAGGGCGAGGGCGAGGGCCGCCCCTACGAGGGCACCCAGACCGCCAAGCTGAAGGTGACCAAGGGCGGCCCCCTGCCCTTCGCCTGGGACATCCTGTCCCCTCAGTTCATGTACGGCTCCAAGGCCTACGTGAAGCACCCCGCCGACATCCCCGACTACTTGAAGCTGTCCTTCCCCGAGGGCTTCAAGTGGGAGCGCGTGATGAACTTCGAGGACGGCGGCGTGGTGACCGTGACCCAGGACTCCTCCCTGCAGGACGGCGAGTTCATCTACAAGGTGAAGCTGCGCGGCACCAACTTCCCCTCCGACGGCCCCGTAATGCAGAAGAAGACCATGGGCTGGGAGGCCTCCTCCGAGCGGATGTACCCCGAGGACGGCGCCCTGAAGGGCGAGATCAAGCAGAGGCTGAAGCTGAAGGACGGCGGCCACTACGACGCCGAGGTCAAGACCACCTACAAGGCCAAGAAGCCCGTGCAGCTGCCCGGCGCCTACAACGTCAACATCAAGCTGGACATCACCTCCCACAACGAGGACTACACCATCGTGGAACAGTACGAGCGCGCCGAGGGCCGCCACTCCACCGGCGGCATGGACGAGCTGTACAAGTAA

**EMCV IRES (gray) and HA (pink) sequences in all plasmid DNA template vectors used for synthesis of IRES dependent HA expressing mRNAs. The start and stop codons are underlined. Sequences used for HA ORF amplification are shown in bold (see primer sequences below):**

CCCCTCTCCCTCCCCCCCCCCTAACGTTACTGGCCGAAGCCGCTTGGAATAAGGCCGGTGTGCGTTTGTCTATATGTTATTTTCCACCATATTGCCGTCTTTTGGCAATGTGAGGGCCCGGAAACCTGGCCCTGTCTTCTTGACGAGCATTCCTAGGGGTCTTTCCCCTCTCGCCAAAGGAATGCAAGGTCTGTTGAATGTCGTGAAGGAAGCAGTTCCTCTGGAAGCTTCTTGAAGACAAACAACGTCTGTAGCGACCCTTTGCAGGCAGCGGAACCCCCCACCTGGCGACAGGTGCCTCTGCGGCCAAAAGCCACGTGTATAAGATACACCTGCAAAGGCGGCACAACCCCAGTGCCACGTTGTGAGTTGGATAGTTGTGGAAAGAGTCAAATGGCTCTCCTCAAGCGTATTCAACAAGGGGCTGAAGGATGCCCAGAAGGTACCCCATTGTATGGGATCTGATCTGGGGCCTCGGTGCACATGCTTTACATGTGTTTAGTCGAGGTTAAAAAAACGTCTAGGCCCCCCGAACCACGGGGACGTGGTTTTCCTTTGAAAAACACGATGATAATATGG**CCACAACCATGAAGGCAAACCTACTGGTCCTGTTATGTGCACTTGCAGCTGCAGATGC**AGACACAATATGTATAGGCTACCATGCGAACAATTCAACCGACACTGTTGACACAGTGCTCGAGAAGAATGTGACAGTGACACACTCTGTTAACCTGCTCGAAGACAGCCACAACGGAAAACTATGTAGATTAAAAGGAATAGCCCCACTACAATTGGGGAAATGTAACATCGCCGGATGGCTCTTGGGAAACCCAGAATGCGACCCACTGCTTCCAGTGAGATCATGGTCCTACATTGTAGAAACACCAAACTCTGAGAATGGAATATGTTATCCAGGAGATTTCATCGACTATGAGGAGCTGAGGGAGCAATTGAGCTCAGTGTCATCATTCGAAAGATTCGAAATATTTCCCAAAGAAAGCTCATGGCCCAACCACAACACAACCAAAGGAGTAACGGCAGCATGCTCCCATGCGGGGAAAAGCAGTTTTTACAGAAATTTGCTATGGCTGACGGAGAAGGAGGGCTCATACCCAAAGCTGAAAAATTCTTATGTGAACAAGAAAGGGAAAGAAGTCCTTGTACTGTGGGGTATTCATCACCCGTCTAACAGTAAGGATCAACAGAATATCTATCAGAATGAAAATGCTTATGTCTCTGTAGTGACTTCAAATTATAACAGGAGATTTACCCCGGAAATAGCAGAAAGACCCAAAGTAAGAGATCAAGCTGGGAGGATGAACTATTACTGGACCTTGCTAAAACCCGGAGACACAATAATATTTGAGGCAAATGGAAATCTAATAGCACCAAGGTATGCTTTCGCACTGAGTAGAGGCTTTGGGTCCGGCATCATCACCTCAAACGCATCAATGCATGAGTGTAACACGAAGTGTCAAACACCCCTGGGAGCTATAAACAGCAGTCTCCCTTTCCAGAATATACACCCAGTCACAATAGGAGAGTGCCCAAAATACGTCAGGAGTGCCAAATTGAGGATGGTTACAGGACTAAGGAACATTCCGTCCATTCAATCCAGAGGTCTATTTGGAGCCATTGCCGGTTTTATTGAAGGGGGATGGACTGGAATGATAGATGGATGGTACGGTTATCATCATCAGAATGAACAGGGATCAGGCTATGCAGCGGATCAAAAAAGCACACAAAATGCCATTAACGGGATTACAAACAAGGTGAACTCTGTTATCGAGAAAATGAACATTCAATTCACAGCTGTGGGTAAAGAATTCAACAAATTAGAAAAAAGGATGGAAAATTTAAATAAAAAAGTTGATGATGGATTTCTGGACATTTGGACATATAATGCAGAATTGTTAGTTCTACTGGAAAATGAAAGGACTCTGGATTTCCATGACTCAAATGTGAAGAATCTGTATGAGAAAGTAAAAAGCCAATTAAAGAATAATGCCAAAGAAATCGGAAATGGATGTTTTGAGTTCTACCACAAGTGTGACAATGAATGCATGGAAAGTGTAAGAAATGGGACTTATGATTATCCCAAATATTCAGAAGAGTCAAAGTTGAACAGGGAAAAGGTAGATGGAGTGAAATTGGAATCAATGGGGATCTATCAGATTCTGGCGATCTACTCAACTGTCGCCAGTTCACTGGTGCTTTTGGTCTCCCTGGGGGCAATCAGTTTCTGGATGTGTTCTAATGGATCTTTGCAGTGCAGAATATGCATCTGA**GATTAGAATTTCAGAAATATGAGGAAAAACACCCTTGTTTCTACTGAGTCGACGAT**

**The PR8 HA protein sequence that was synthetized in transfected myocytes and MDCK cells *in vitro* and in vaccinated mice *in vivo*. The first 4 amino acids (MATT, highlighted in blue) were added to the HA sequence because this sequence is required for EMCV IRES dependent expression:**

MATTMKANLLVLLCALAAADADTICIGYHANNSTDTVDTVLEKNVTVTHSVNLLEDSHNGKLCRLKGIAPLQLGKCNIAGWLLGNPECDPLLPVRSWSYIVETPNSENGICYPGDFIDYEELREQLSSVSSFERFEIFPKESSWPNHNTTKGVTAACSHAGKSSFYRNLLWLTEKEGSYPKLKNSYVNKKGKEVLVLWGIHHPSNSKDQQNIYQNENAYVSVVTSNYNRRFTPEIAERPKVRDQAGRMNYYWTLLKPGDTIIFEANGNLIAPRYAFALSRGFGSGIITSNASMHECNTKCQTPLGAINSSLPFQNIHPVTIGECPKYVRSAKLRMVTGLRNIPSIQSRGLFGAIAGFIEGGWTGMIDGWYGYHHQNEQGSGYAADQKSTQNAINGITNKVNSVIEKMNIQFTAVGKEFNKLEKRMENLNKKVDDGFLDIWTYNAELLVLLENERTLDFHDSNVKNLYEKVKSQLKNNAKEIGNGCFEFYHKCDNECMESVRNGTYDYPKYSEESKLNREKVDGVKLESMGIYQILAIYSTVASSLVLLVSLGAISFWMCSNGSLQCRICI*

**Sequences of DNA primers used for amplification of the HA ORF fragment. The fragment was amplified using Influenza A/PR/8/34 virus genomic RNA isolation by RT-PCR. The PCR insert was specifically designed for suitable MscI - EcoRV ligation into EMCV IRES harboring DNA template that was later used for HA-mRNA synthesis. The same A/PR/8/34 virus was used for mouse challenge experiments to insure an antigen match:**

CCACAACCATGAAGGCAAACCTACTGGTCCTGTTATGTGCACTTGCAGCTGCAGATGC (forward)

ATCGTCGACTCAGTAGAAACAAGGGTGTTTTTCCTCATATTTCTGAAATTCTAATC (reverse)
